# Global regulation of methane emission from natural lakes

**DOI:** 10.1038/s41598-018-36519-5

**Published:** 2019-01-22

**Authors:** Lúcia Fernandes Sanches, Bertrand Guenet, Claudio Cardoso Marinho, Nathan Barros, Francisco de Assis Esteves

**Affiliations:** 1 Laboratório de Limnologia, Federal University of Rio de Janeiro, 68020 Rio de Janeiro, Brazil; 2 Laboratoire des Sciences du Climat et de l’Environnement, LSCE/IPSL, CEA-CNRS-UVSQ, Université Paris-Saclay, F-91191 Gif-sur-Yvette, France; 3 Federal University of Juiz de Fora, Juiz de Fora, 36036-900 Brazil

**Keywords:** Carbon cycle, Limnology

## Abstract

Methane (CH_4_) emissions from lakes are the largest of the emissions from freshwater ecosystems. We compile open water CH_4_ emission estimates from individual lakes from all over the world and consider the three main emission pathways: diffusive; ebullitive; and storage. The relationships between emissions, environmental variables, lake characteristics and methodological approaches are investigated for the measurements from 297 lakes. We show that environmental factors, such as temperature and precipitation, act as important driving factors for CH_4_ emissions, with higher emissions occurring where air temperature and precipitation are high. The diffusive flux of CH_4_ was found to be positively related to dissolved organic carbon concentration. Diffusive flux is the most frequently estimated component of the total flux, while the other emission pathways are often neglected. Based on the cases where all three components of the total flux were measured (30 lakes), we estimate that measuring the diffusive emission only, and then assuming that the value obtained is a good surrogate for the total emission, would have led to a 277% underestimation of the real total flux. In addition we show that the estimation of fluxes is method-dependent with substantial differences revealed between the flux estimates obtained from different measurement techniques. Some of this uncertainty is due to technical constraints which should not be neglected, and lake CH_4_ flux measurement techniques require thorough re-evaluation.

## Introduction

Freshwater ecosystems are significant sources of methane (CH_4_), and natural lakes contribute approximately 70% of the freshwater CH_4_ emission, which is disproportionate to the small area they occupy^1^. CH_4_ production in lakes mainly occurs from anoxic sediment^2–4^, and can reach the atmosphere via four emission pathways: ebullitive flux, diffusion, storage flux and emissions from aquatic vegetation. The diffusive flux and, to a lesser extent, ebullition are the most studied of these emission pathways to date^1^.

Ebullition is considered to be the most important contributor to emissions from surface waters^1^, especially in shallow lakes (<20 m). Released bubbles of CH_4_ correspond to a direct vertical flux from the anoxic sediment to the atmosphere, with little physical or chemical loss or any biological interaction within the water column^5^. A large proportion of CH_4_ within the water column is oxidized by bacteria^6^ with oxidation rates varying from between 15 to 90%^7,8^. CH_4_ that escapes oxidation and reaches the upper mixed layer of the water column is then emitted by diffusive flux. Storage flux occurs when dissolved CH_4_, which has accumulated in the water column during periods of stratification, when CH_4_ builds up in the anoxic layer, or when ice formation prevents gas exchanges with the atmosphere, is suddenly emitted by diffusion. These sudden emissions may be triggered by lake overturn^9,10^ or by melting of ice^11,12^. Finally, the importance of CH_4_ flux through emergent macrophytes is directly related to the presence, abundance and morpho-physiological characteristics of marginal vegetation in the aquatic ecosystem^13^. It is the only emission pathway that doesn’t originate directly from the so-called “open water” parts of the lakes.

Due to the increasing atmospheric concentration of CH_4_ and its impact on climate^14,15^, CH_4_ emissions from lakes have become more and more frequently measured^1,16–18^ in recent years. The main focus of these studies has been to establish values of emission per unit area and time^19–22^, but the estimates generally include only one or two emission pathways, assuming the other fluxes to be negligible. This assumption limits the possibility of accurately scaling up the values to estimate total CH_4_ emission rates from lakes at a regional or global scale^23^. Additionally, the drivers that can influence the total flux, or each individual pathway, are not well understood, although some environmental and methodological driving factors have been identified^23–25^. Temperature and precipitation, for example, were suggested as important regulators for CH_4_ emissions during one modeling exercise^24^. Lake area, water depth, the concentrations of total phosphorous and dissolved organic carbon have also been proposed as regulators for CH_4_ emissions at high latitudes^23^. Finally, the impact of methodological and biophysical drivers has not been assessed at regional or global scales and estimates of CH_4_ emissions at these scales still have large uncertainties.

Determining the environmental and methodological factors that are most related to the dynamics of each different flux can provide an important basis to predict and to understand the large differences in CH_4_ emissions that occur between different lakes and between different compartments of the same ecosystem. Thus, this study aims to determine the driving factors related to each CH_4_ emission pathway from the open waters of lakes at a large scale. Additionally, we aim to quantify the uncertainties associated with the assumption that some emission pathways can be neglected.

## Results

### Climate and landscape: importance of external drivers

**Figure 1:**
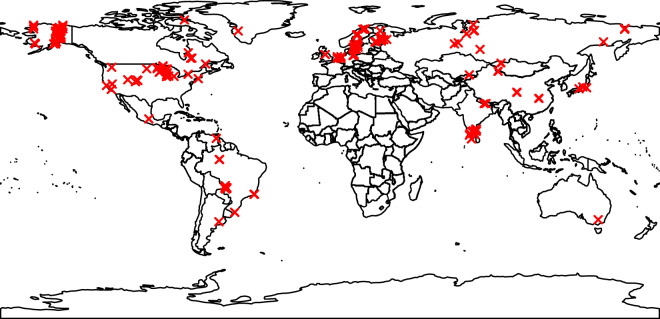
Map showing the location of the lakes. The map was created using R. 3.2.3 (https://www.R-project.org/). The rworldmap packages was used to draw the map (http://cran.r-project.org/web/packages/rworldmap).

**Figure 2:**
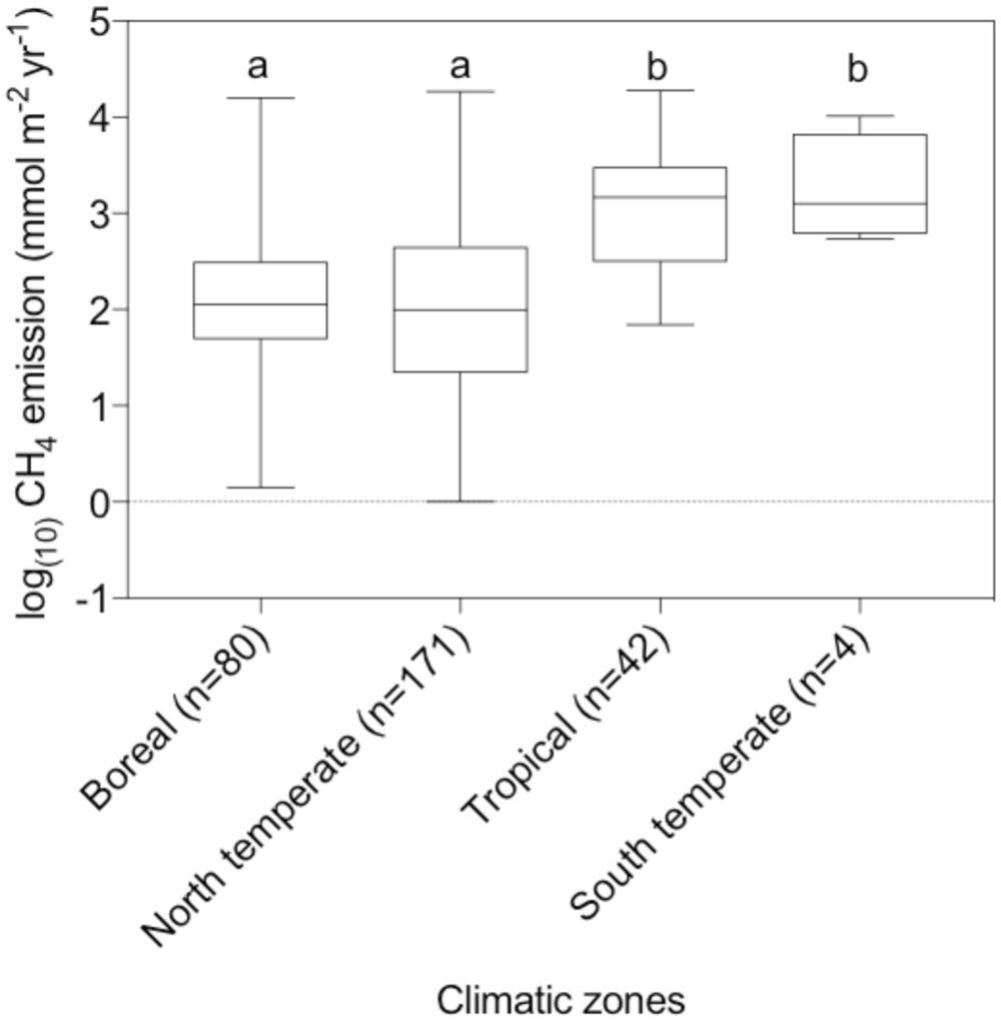
Boxplot of methane emission estimates for each climatic zone.

**Figure 3:**
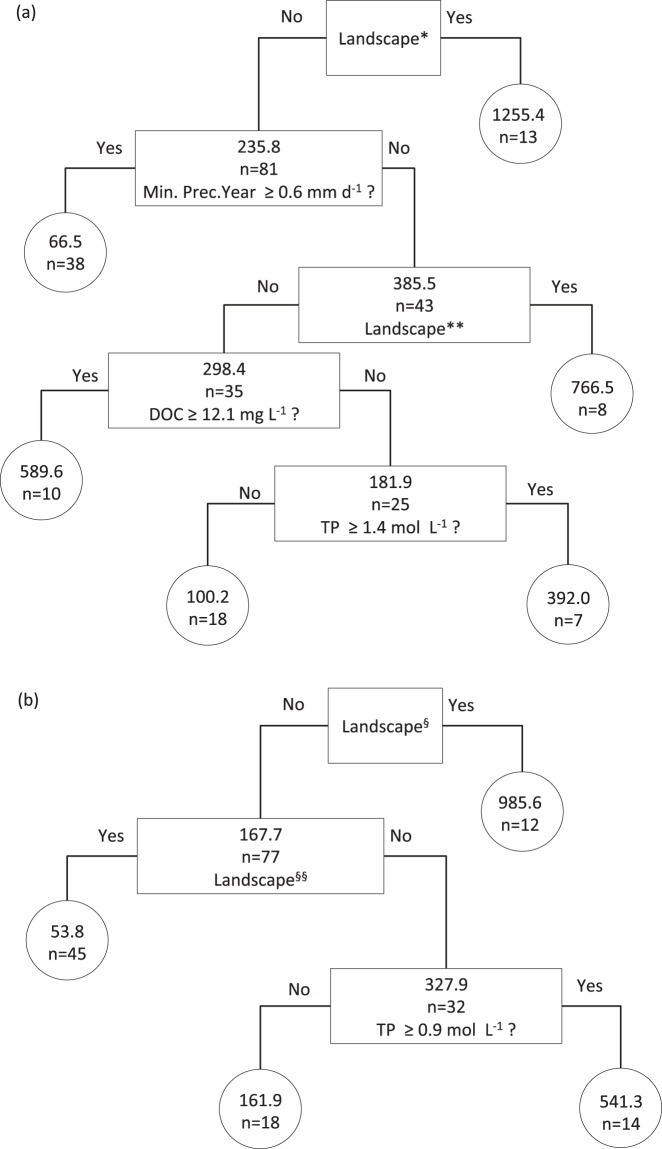
Regression trees performed on the CH_4_ emissions estimated for the second subgroup of lakes (see SI): (**a**) Total flux and (**b**) Diffusive flux. Each split in the tree represents a yes or no answer to the question shown in the node box. The nodes also list the mean values of CH_4_ flux and the number of lakes (n). The circles are terminal nodes. Landscape = Is the landscape around the lake composed by: *needle-leaved evergreen tree cover + cultivated and managed areas, broadleaved deciduous tree cover or closed-open evergreen shrub cover; **closed-open herbaceous cover, cultivated and managed or a mosaic; ^§^broadleaved deciduous tree cover or closed-open evergreen shrub cover; ^§§^Sparse herbaceous or sparse shrub, mosaic, closed-open herbaceous cover or snow? Min. monthly precipitation = annual mean of minimum monthly precipitation during the sampling year (mm d^−1^). DOC = dissolved organic carbon. TP = total phosphorous.

Based on data from 297 lakes (Fig. 1), we observed that CH_4_ emissions were significantly different across climatic zones (p < 0.001, Fig. 2). The lowest values were found in boreal and north temperate zones whilst higher values were found in the tropics and the south temperate zones (Fig. 2). The statistical difference between the values for different climatic zones was found for all CH_4_ emission pathways (p < 0.05 for storage fluxes, p < 0.001 for diffusive, total, and ebullitive fluxes, Table S1). Total fluxes of CH_4_ were found to be significantly and negatively correlated with minimum monthly air temperature (p < 0.01; Table S1) when it was considered as a single factor. Total fluxes of CH_4_ were also significantly correlated with minimum and maximum monthly air temperature when analyzed in interaction with other variables (Table S1). Diffusive fluxes of CH_4_ were significantly and positively correlated with minimum and maximum monthly air temperature (p < 0.001) and with all the temperature-related variables when analyzed in interaction with other variables (Table S1). The ebullition and storage fluxes of CH_4_ were also significantly correlated with minimum monthly air temperature (p < 0.05 and p < 0.001, respectively) but higher minimum monthly air temperature was found to increase ebullition whereas the opposite effect was found for storage flux. Precipitation related variables were also important driving factors, mainly for diffusive and storage fluxes. Storage flux significantly decreased with increasing annual average precipitation (p < 0.001). Finally, the regression tree analysis performed for the total and diffusive flux on the second subgroup (i.e. those for which information on dissolved organic carbon (DOC), phosphorus concentration (TP) and maximum depth was available) demonstrated that precipitation was an important driver of CH_4_ emissions (Fig. 3).

Analysis of the first subgroup of lakes showed that the surrounding landscape significantly affected all of the CH_4_ emissions pathways except for storage (p < 0.001). Regression tree analysis for the total and diffusive fluxes highlights the importance of the surrounding landscape as a driving factor for these CH_4_ emission pathways (Fig. 3b). However, this result should be treated with caution. The classification of lakes by landscape type often tends to group together lakes which lie within the same geographical area, and so the landscape effect we observed might just be an artifact created by this grouping.

### The importance of lake characteristics

The analysis of the first subgroup which consists of all lakes for which there is information on area, indicated that area, when considered as a single factor, was only an important variable for ebullition and the total flux (p < 0.01 and p < 0.05, respectively Table S1). Nevertheless, significant interactions (p < 0.001) were observed between area and landscape, or between area and average temperature, for diffusive flux. A significant interaction between area and landscape was also observed (p < 0.001) for ebullition.

**Figure 4:**
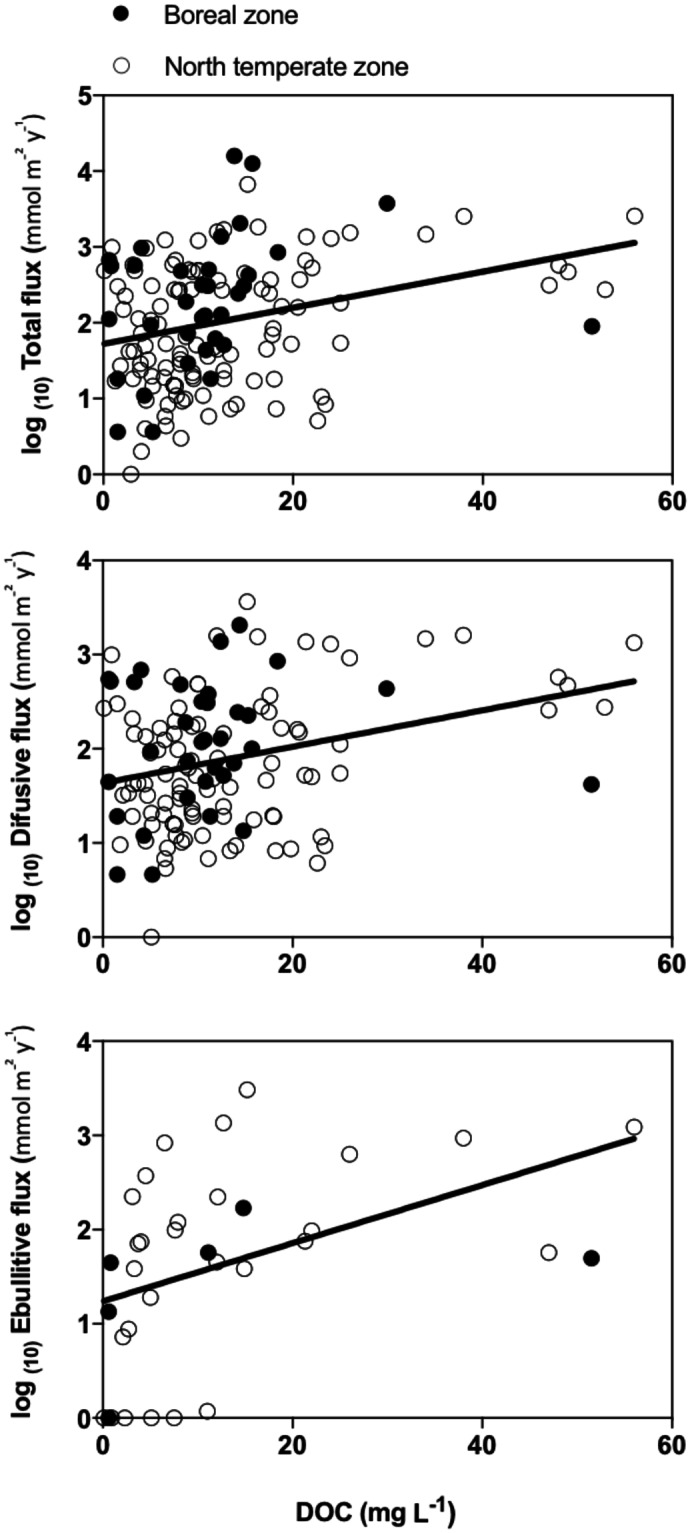
Relationships between the CH_4_ flux and dissolved organic carbon (DOC, mg C L^−1^) for the second subgroup of lakes (see SI): (a) total flux; (b) diffusive flux; (c) ebullitive flux. The total flux is the sum of the different fluxes measured for each lake.

Through analysis of the second subgroup of lakes, for which DOC, TP and depth are considered as explanatory variables, DOC was highlighted as an important driving factor for total and diffusive CH_4_ emissions (Table S1). The maximum depth was another variable related to lake characteristics selected by the models for total, and diffusive fluxes of CH_4_ (Table S1), with fluxes found to be positively correlated with it. Total phosphorous, a proxy for eutrophication, only significantly affected the flux when in combination with other variables and then only for the diffusive flux. The regression tree (Fig. 3b) highlights the importance of total phosphorous as a lake characteristic influencing the values of emission, with higher emissions via diffusion occurring when total phosphorous content was high. DOC was always positively correlated with emissions either as single factor or in interaction, except for case of storage (Fig. 4).

### CH_4_ emission and its dependency on the methodology

**Figure 5:**
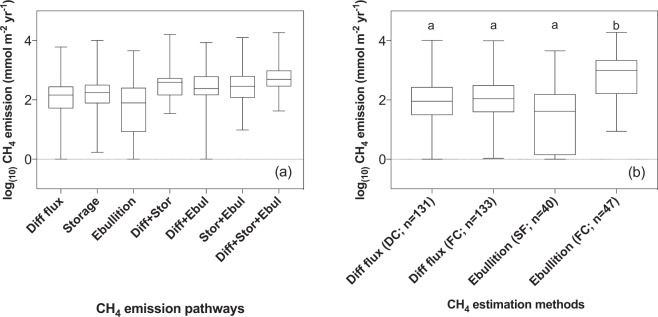
Boxplot of methane emission estimates presented according to estimation method and emission pathway. (**a**) Contribution of each flux separately and all possible combinations to open water CH_4_ emission when all three types of flux were measured (n = 24). Diff. Flux = diffusive flux, Stor. = storage, Ebul. = ebullition. Bars combining the three fluxes represent the real total flux of an ecosystem. (**b**) Differences between CH_4_ fluxes (diffusive and ebullitive) measured using different methods. DC = dissolved concentration; SF = submersed funnel and FC = floating chamber. Different letters above the boxes mean there is a significant difference.

The diffusive flux was the most frequently estimated pathway in the studies used to compile the database (Table S2) and was, therefore, the pathway most used (both individually and as part of another flux component) to estimate the total flux. The ebullitive flux, on the other hand, was the pathway estimated least often. However, for the cases where all three emission pathways were estimated for the same lake, all the pathways made important contributions to the total flux, with 30.9% coming from the diffusive flux, 35.7% from the storage flux and 33.4% from ebullition (Fig. 5a).

The use of different methods to estimate the same CH_4_ emission pathway appeared to be a source of significant differences in total CH_4_ flux estimation. Using the methods as explanatory variables in the linear models, we found significant statistical differences (p < 0.001) between the different methods used to estimate ebullitive fluxes. Ebullitive fluxes estimated by the submerged funnel gas method, are 6 times lower on average than those measured with floating chambers **(**Fig. 5b).

## Discussion

**Figure 6:**
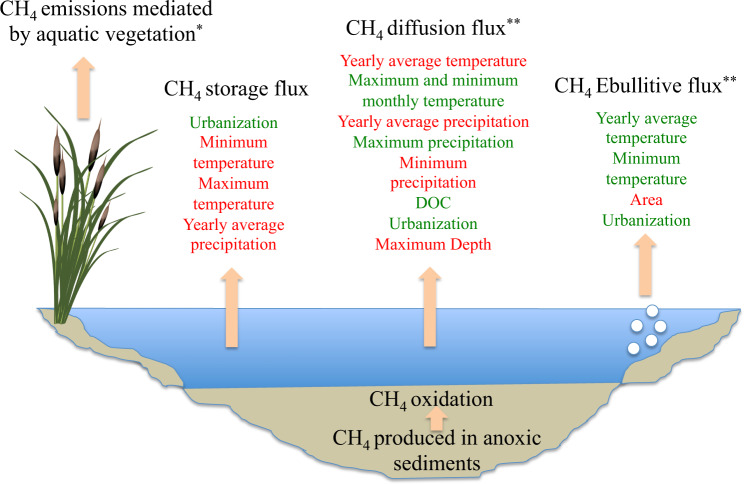
The different emission pathways and the main drivers. Green driver names indicate a positive correlation whereas red indicates a negative correlation. The correlation refers to the analysis done with the second subset of lake data. *Not analyzed in this study. **Significantly affected by the methods used to measure the flux.

Here, we showed that CH_4_ emissions from lakes are correlated to several environmental drivers such as climate related variables (Fig. 6). We also observed different emission patterns in different climatic zones. For example, we found that higher emission rates were reported in climatic zones with higher mean air temperatures (i.e. the tropics and south temperate zones Fig. 2). Therefore, the observed latitudinal pattern of emission is probably related to temperature^26^. This temperature effect is probably related to the role of temperature as a methanogenesis driving factor^27,28^. Additionally, we found that precipitation significantly affected the diffusive and storage emissions of CH_4_. This effect is probably due to the impact of precipitation on lake mixing. These important climate dependencies were not found when different aquatic ecosystems, including lakes, estuaries, oceans, rivers and wetlands were all analyzed together^29^. The absence of any climate dependency in that study is probably explained by the large variations in the CH_4_ emission data, which are reduced when the analysis is restricted to natural lakes only. CH_4_ emissions from natural wetlands have been linked to variations in environmental factors such as temperature and precipitation^24^. Furthermore, higher precipitation generally induces higher runoff and drainage, which impact the local dissolved organic carbon concentration and, ultimately, the lateral fluxes^30^. We may therefore expect higher external inputs, mainly of organic substance, in areas with high precipitation.

Total phosphorous concentration seems to be a factor leading to differences in CH_4_ emissions (Table S1), but its importance is mainly restricted to total and diffusive emissions (Fig. 3). Lakes with high total phosphorous concentration appear to be major sources of CH_4_ emissions. According to the regression tree analysis, the diffusion flux in lakes with total phosphorous concentration higher than 0.9 mol L^−1^ was 5 times higher than in lakes with total phosphorous concentration less than 0.9 mol L^−1^. This relationship may be significant as currently the major impacts of human activities affecting lakes are increases in nutrients and organic matter concentration^18,31,32^. If these factors impact lake CH_4_ dynamics, they can be important drivers of global change in aquatic ecosystems^33^.

Eutrophication, caused by anthropogenic sources is caused by a large input of limiting nutrients and a consequent increase in primary production. The subsequent sinking and decay of algal blooms can have direct effects on methanogenesis - providing a greater supply of substrate - as well as indirect effects, due to a reduction in dissolved oxygen concentration, especially in the hypolimnion^34^. Thus the trophic state impacts CH_4_ concentrations in water and sediment^16,34–36,37^ as well as the CH4 emission to the atmosphere^16,18^. However, it is important to note that lakes grouped by the first split in Fig. 3 are mainly from the same region. Consequently, the high levels of CH_4_ emission observed in this group might be due to other, separate, environmental characteristics that are specifically related to this region.

The fact that area was a significant explanatory variable, mainly when interactions with other variables such as landscape or average precipitation are considered, suggests that the effects of some factors driving CH_4_ emission might vary according to lake area. In particular, the significant interactions between landscape and area suggest that larger lakes are less sensitive to the effect of landscape than smaller lakes. Furthermore, large ebullitive emissions are reported in the littoral zone, which represents the shallower part of lakes^23,38–40^.

In our database, DOC was an important driver of the diffusive emissions, with the relationship between DOC and CH_4_ emission always being positive (Fig. 4, Table S1). This relationship is consistent with the available literature, showing that DOC is an indicator of the substrate availability for methanogenesis^41–43^. DOC may come from autochthonous sources (algae exudation, microbial decomposition products) or from allochthonous sources (terrestrial organic matter). Both sources are substrate for methanogenesis^33,44^. Anthropogenic activities may also increase DOC leaching from soil to aquatic ecosystems^32,45,46^, which can represent an additional source of energy for microbial populations^47,48^. As terrestrial DOC supply can increase methanogenesis rates^33^, it could also influence CH_4_ emission rates.

The uncertainties on the different emission pathways are considerable and we have identified some aspects of the methodology which can help to explain such large variability. The large imbalance in CH_4_ flux measurements between diffusion and the other mechanisms might be due to the development of easy-to-use techniques for measuring the diffusion flux. These techniques make use of mathematical equations involving the gas concentration in the water column and in the air, and other environmental variables such as water temperature and wind speed to estimate the flux^49^. Since it was first proposed, this simple, indirect measurement method has been widely applied^21,23,28^. Currently, we also have the possibility of making direct field measurements of CH_4_ diffusive emissions. This is mainly done with the use of floating chambers, which is inexpensive and simple^50^, and has become the most common technique for measuring gas fluxes in lakes^1,10,19^. However, the exact details of the implementation of the method can vary extensively.

The observation that different estimates of CH_4_ emissions are obtained when different measurement methods are applied is in agreement with a previous finding at Lake Rostee in Switzerland^25^ and at a lake in Finland^51^, where lower estimates for diffusive flux were obtained using the indirect approach (i.e. CH_4_ dissolved concentration in water column). It should be noted that, in our case, when the two lake groups are divided according to the estimation method used, there is also a distinction between the two groups in terms of depth. Lakes for which the CH_4_ emission flux was measured directly were, on average, shallower. Results which appeared to be related to the sampling method used might, instead, have been related to the lake depth. Indeed, shallow lakes do generally present higher CH_4_ emission rates^23^. We also observed a significant effect of the method used to estimate ebullition flux on the values obtained. In this case the effect might be due to the fact that the floating chambers used to estimate ebullition flux also captured the diffusion flux.

We showed that, when the three emission pathways were estimated for the same lake, storage and ebullition, which were the least often measured pathways of CH_4_ emission, were the main contributor to the total flux. The diffusive flux was the least important contributor to the total flux, although it was the CH_4_ emission pathway estimated most often. The assumption that measuring only the diffusion flux is a good surrogate for the total flux^23^ may lead to significant underestimates of the CH_4_ flux to the atmosphere. For instance, in lakes where all three pathways were estimated, assuming that the pathways other that diffusion are negligible would lead to an underestimation of the total flux of up to 277%.

The lack of ebullition and storage flux measurements may be closely linked to both emission characteristics and technical difficulties. Emissions by bubbles and storage flux have high temporal variability and, in contrast to the diffusive flux, cannot be measured continuously. According to our database, the ebullitive flux was the least often measured component, even though it was the main contributor towards the total flux. It should be noted that storage flux only occurs in a limited number of lakes as it requires water column stratification or surface layer freezing. Ebullition is a much more commonly occurring process, since most of the lakes of the world are shallow^52^.

Diffusion, the most commonly estimated pathway of CH_4_ emission (265 in a total of 297 estimates), makes a limited contribution to the total flux, highlighting the importance of measuring all the other emission pathways despite the technical challenges involved. Additionally, systematic measurements of environmental variables and lake characteristics, such as DOC, TP, etc., that are closely related to the CH_4_ emissions of lakes, would make it easier to investigate the mechanisms that influence CH_4_ emission from lakes at regional or global scales. Finally, if we are to be able to explain the substantial differences found in the emission fluxes between different lakes and accurately estimate the CH_4_ fluxes from lake surfaces to the atmosphere, the methods used to measure CH_4_ fluxes require thorough evaluation.

## Methods

### Database

A database was created by compiling estimates of the CH_4_ emission from lakes of different climatic zones around the world using “CH_4_” or “methane” and “lake” or “wetland” as keywords in the web of knowledge in January and February 2018. Unpublished data of estimates of diffusive flux from 5 tropical coastal lagoons available in the thesis document of Marinho 2013 from the Institute of Biophysics Carlos Chagas Filho at the Federal University of Rio de Janeiro (Table S1) were also included in the database. We gathered data from the literature for each ecosystem, using studies which reported the contribution of at least one of the CH_4_ fluxes from open waters (i.e. diffusive flux, ebullition and storage). We chose studies for which data from individual lakes could be extracted and we therefore excluded studies which present only average values for groups of lakes. In total, there were 55 studies with data from 297 lakes (Fig. 1), published between 1977 and 2017, which clearly separate the different flux components (Table S2). To extract data from published figures, we used the free software Engauge Digitizer 4.1. CH_4_ emission estimates are generally reported in studies as daily or annual rates, and less often as hourly fluxes. When the CH_4_ flux was measured as a daily rate we extrapolated the data to estimate an annual rate. Extrapolation of daily CH_4_ emissions into an annual rate may create large uncertainties if the sampling period is not representative. Nevertheless, this method has been successfully used in previous studies^1^.

Here, we use the term “total flux” to represent the sum of the measured fluxes for each lake. This number can be an underestimate of the actual total flux since most of the studies consider only one or two of the emission pathways. Of the 297 lakes included in the database, all three flux components were measured for only 30 lakes.

### Ancillary data

To better understand the factors controlling each of the different CH_4_ emission pathways, several variables were collected. The geographic coordinates, area, maximum depth, DOC and TP were obtained from each study or from related studies on the same lake. Information on the landscape surrounding each lake, including the type of vegetation cover and/or land use, was obtained either directly from the relevant study, or from the global land cover 2000 database^53^. The distinction between managed and unmanaged areas was based on the global land cover 2000 database classification by considering that “artificial surfaces” and “cultivated and managed areas” where the two managed classes. Climate-related information for each lake (minimum, maximum and average monthly precipitation and air temperature) was obtained by using the relevant datapoints from the global climate data set CRUNCEP v5 (Viovy *et al*., pers. com.) at a resolution of 0.5° × 0.5°. This data set combines the CRU-TS2^54^ monthly climatology, covering the period 1901–2013, with the NCEP reanalysis, starting from 1948. The climatic zones were defined based on the latitude of each lake; boreal if higher than 60°N; north temperate between 30°N and 60°N; tropical between 30°S and 30°N; and south temperate between 60°S and 30°S.

Four categorical variables related to the methods used to estimate the CH_4_ fluxes were added, as follows. The variable “estimation component” describes the emission pathways that were used to estimate the total CH_4_ flux. The variable “estimation method”, describes the method used to estimate CH_4_ emissions: floating chamber and dissolved CH_4_ concentration for diffusive and storage fluxes; floating chambers and bubble traps for ebullitive emission. Thus, the total emission estimated by a given study might involve a different combination of methods than the total emission estimated another study. The variable “starting year” indicates when the lakes were sampled, and the variable “time in months” provides the length of the sampling events. These temporal aspects are important because CH_4_ emissions are temporally dynamic with occasional episodes of high emissions which might not be representative of the annual rates^18^.

### Statistical methods

The relationships between CH_4_ emission and lake characteristics, environmental variables and methodological approaches were investigated using two statistical methods: Stepwise linear regression, and regression tree analysis using the software R 3.0.2. The regression trees provide a complementary, non-parametric approach^55^ and are used here to rank the importance of the lake characteristics on CH_4_ emission. To reduce the heterogeneity of the variances and meet the assumptions of multiple linear regression, the data were log-transformed prior to performing the stepwise regressions.

Both statistical methods were applied first on 293 lakes for total flux (four were excluded from the complete database because of missing information), diffusion was measured over 261 lakes, ebullition over 93 lakes and storage flux over 87. In this first step, to ensure we were analyzing the most complete database, we did not use explanatory variables related to lake characteristics and landscape, but only the following explanatory variables: estimation component; method of estimation; time in months; starting year; climatic zone; and climatic information data. For subsequent analyses, to help understand the factors controlling CH_4_ emissions, the database was split into two subgroups. The first subgroup consisted of all lakes for which there was information on area (205, 176, 55 and 93 lakes for total, diffusive, ebullitive and storage fluxes, respectively). The second subgroup included all lakes for which there was information on DOC, TP and maximum depth (95, 89, 49 and 27 lakes for total, diffusive, ebullitive and storage fluxes, respectively). For the second subgroup, ebullition flux was excluded from the analysis, as there were not enough DOC concentration data available. The database and the scripts are available upon request to the corresponding author.

## Electronic supplementary material

Supplementary material
