# Author Correction: Global regulation of methane emission from natural lakes

**DOI:** 10.1038/s41598-021-00321-7

**Published:** 2021-10-25

**Authors:** Lúcia Fernandes Sanches, Bertrand Guenet, Claudio Cardoso Marinho, Nathan Barros, Francisco de Assis Esteves

**Affiliations:** 1 Laboratório de Limnologia, Federal University of Rio de Janeiro, Rio de Janeiro, 68020 Brazil; 2 Laboratoire des Sciences du Climat et de l’Environnement, LSCE/IPSL, CEA-CNRS-UVSQ, Université Paris-Saclay, 91191 Gif-sur-Yvette, France; 3 Federal University of Juiz de Fora, Juiz de Fora, 36036-900 Brazil

Correction to: *Scientific Reports* 10.1038/s41598-018-36519-5, published online 22 January 2019

The original version of this Article contained errors.

In the Results section, under the subheading ‘CH_4_ emission and its dependency on the methodology’,

“However, for the cases where all three emission pathways were estimated for the same lake, all the pathways made important contributions to the total flux, with 36.0% coming from the diffusive flux, 38.8% from the storage flux and 27.2% from ebullition (Fig. 5a).”

now reads:

“However, for the cases where all three emission pathways were estimated for the same lake, all the pathways made important contributions to the total flux, with 30.9% coming from the diffusive flux, 35.7% from the storage flux and 33.4% from ebullition (Fig. 5a).”

In addition, in the legend of Figure 5,

“(**a**) Contribution of each flux separately and all possible combinations to open water CH_4_ emission when all three types of flux were measured (n = 15).”

now reads:

“(**a**) Contribution of each flux separately and all possible combinations to open water CH_4_ emission when all three types of flux were measured (n = 24).”

The original Article has been corrected.

