# Supplementary material for: Global regulation of methane emission from natural lakes
Source: Sci Rep. 2019 Jan 22;9:255. doi: 10.1038/s41598-018-36519-5 (PMC6343025; doi:10.1038/s41598-018-36519-5)
Supplement: Supplementary file 1 — Supplementary material [file 41598_2018_36519_MOESM1_ESM.docx]

Global regulation of methane emission from natural lakes

Lúcia Fernandes Sanches, Bertrand Guenet, Claudio Cardoso Marinho, Nathan Barros, Francisco de Assis Esteves

Includes:

Supporting Table 1: Stepwise regression results for different types of CH_4_ emission.

Supporting Table 2: Source and extent of the lake data.

**Supporting information**

Supporting Table 1: Stepwise regression results for different types of CH_4_ emission. 1) “Complete lakes database” with variables available for most of the lakes; 2) First group of lakes with available information on Area; 3) Second group of lakes with available information on DOC, TP and Maximum depth. The symbol * means interaction between variables.

| CH_4_ emissions | 1. Complete database | | | | 1. Data of Area | | | | 1. Data of DOC, TP and Max. Depth | | | |
| --- | --- | --- | --- | --- | --- | --- | --- | --- | --- | --- | --- | --- |
|  | n | Significant Variables | p | R^2^ | n | Significant Variables | p | R^2^ | n | Significant Variables | p | R^2^ |
| Total flux | 293 | Estimation Component | <0.0001 | 0.83 | 205 | Area | 0.04 | 0.94 | 95 | Climatic Zone | 0.017 | 0.89 |
|  |  | Estimation Method | <0.0001 |  |  | Estimation Method | <0.0001 |  |  | Estimation Component | <0.0001 |  |
|  |  | Climatic Zone | <0.0001 |  |  | Estimation Component | <0.0001 |  |  | Duration | <0.0001 |  |
|  |  | Min. Temperature | 0.009 |  |  | Landscape | <0.0001 |  |  | Year Precipitation | <0.0001 |  |
|  |  | Estimation Component* Climatic Zone | 0.002 |  |  | Climatic Zone | <0.0001 |  |  | Landscape | <0.0001 |  |
|  |  | Estimation Component* Max. Temperature | <0.0001 |  |  | Max. Temperature | 0.003 |  |  | DOC | 0.046 |  |
|  |  | Estimation Component *  Average Temperature | 0.0009 |  |  | Stating year | <0.0001 |  |  | Max. Depth | 0.039 |  |
|  |  | Estimation Method*Min Temperature | 0.009 |  |  | Duration | 0.02 |  |  | Climatic Zone*  DOC | <0.0001 |  |
|  |  | Estimation Method*Max Temperature | <0.0001 |  |  | Year Precipitation | 0.002 |  |  | Estimation Component* Year Precipitation | 0.019 |  |
|  |  | Estimation Method* Starting year | 0.048 |  |  | Area* Estimation Method | 0.003 |  |  | Starting Year *Max. Depth | 0.008 |  |
|  |  | Climatic Zone*Min. Temperature | <0.0001 |  |  | Area* Estimation Component | 0.015 |  |  |  |  |  |
|  |  | Climatic Zone*Max. Temperature | 0.049 |  |  | Area* Landscape | 0.0003 |  |  |  |  |  |
|  |  | Climatic Zone*  Starting year | 0.005 |  |  | Area*Climatic Zone | 0.0018 |  |  |  |  |  |
|  |  | Min. Temperature*  Average Temperature | <0.0001 |  |  | Area*Max. Temperature | 0.0006 |  |  |  |  |  |
|  |  | Min. Temperature* Year Precipitation | <0.0001 |  |  | Area*Average Temperature | <0.0001 |  |  |  |  |  |
|  |  | Average Temperature*Starting year | 0.041 |  |  | Area* Year Precipitation | 0.014 |  |  |  |  |  |
|  |  |  |  |  |  | Estimation Method* Estimation Component | 0.002 |  |  |  |  |  |
|  |  |  |  |  |  | Estimation Method*Landscape | <0.0001 |  |  |  |  |  |
|  |  |  |  |  |  | Estimation Method*Min. Temperature | 0.003 |  |  |  |  |  |
|  |  |  |  |  |  | Estimation Method*Max. Temperature | <0.0001 |  |  |  |  |  |
|  |  |  |  |  |  | Estimation Method*Max. Precipitation | 0.019 |  |  |  |  |  |
|  |  |  |  |  |  | Estimation Component*Climatic Zone | 0.023 |  |  |  |  |  |
| Diffusive flux | 260 | Climatic Zone | <0.0001 | 0.69 | 175 | Estimation Method | <0.0001 | 0.93 | 89 | Climatic Zone | 0.002 | 0.85 |
|  |  | Min. Temperature | <0.0001 |  |  | Landscape | <0.0001 |  |  | Duration | 0.001 |  |
|  |  | Average Temperature | 0.001 |  |  | Climatic Zone | <0.0001 |  |  | Year Precipitation | <0.0001 |  |
|  |  | Max. Precipitation | 0.0002 |  |  | Max. Temperature | 0.002 |  |  | Max Precipitation | 0.0009 |  |
|  |  | Min. Precipitation | 0.04 |  |  | Average Temperature | <0.0001 |  |  | Average Temperature | <0.0001 |  |
|  |  | Estimation Method*Climatic Zone | <0.0001 |  |  | Starting Year | <0.0001 |  |  | Min. Temperature | 0.02 |  |
|  |  | Estimation Method*Max. Temperature | <0.0001 |  |  | Duration | <0.0001 |  |  | DOC | 0.008 |  |
|  |  | Estimation Method*Average Temperature | <0.0001 |  |  | Max. Precipitation | 0.0006 |  |  | Max. Depth | 0.019 |  |
|  |  | Estimation Method*Duration | 0.04 |  |  | Area*Landscape | <0.0001 |  |  | Climatic Zone*Year Precipitation | 0.0003 |  |
|  |  | Estimation Method*Year Precipitation | 0.004 |  |  | Area*  Climatic Zone | <0.0001 |  |  | Duration*DOC | <0.0001 |  |
|  |  | Climatic Zone*Min. Temperature | 0.0019 |  |  | Area*  Max. Temperature | <0.0001 |  |  | Duration*Phosphorous | 0.00016 |  |
|  |  | Climatic Zone*Starting Year | <0.0001 |  |  | Area*Average Temperature | <0.0001 |  |  | Year Precipitation*DOC | 0.003 |  |
|  |  | Climatic Zone*Year Precipitation | 0.0002 |  |  | Area*Duration | 0.009 |  |  | DOC*Phosphorous | 0.019 |  |
|  |  | Min. Temperature*Max. Temperature | 0.011 |  |  | Estimation Method*Min. Temperature | <0.0001 |  |  | Max. Precipitation*Max. Temperature | <0.0001 |  |
|  |  | Min. Temperature*Average Temperature | <0.0001 |  |  | Estimation Method*Average Temperature | <0.0001 |  |  | Min. Precipitation*DOC | 0.0003 |  |
|  |  | Min. Temperature*Max Precipitation | <0.0001 |  |  | Landscape*Max. Temperature | <0.0001 |  |  | Min. Precipitation*Max. Depth | 0.04 |  |
|  |  | Max. Temperature* Starting Year | 0.0008 |  |  | Landscape*Average Temperature | <0.0001 |  |  | Starting Year*Average Temperature | 0.03 |  |
|  |  | Max. Temperature*Duration | 0.0019 |  |  | Landscape* Starting Year | 0.0014 |  |  |  |  |  |
|  |  | Max. Temperature*Year Precipitation | <0.0001 |  |  | Landscape*Duration | 0.005 |  |  |  |  |  |
|  |  | Average Temperature*Duration | <0.0001 |  |  | Landscape*Year Precipitation | 0.005 |  |  |  |  |  |
|  |  | Average Temperature*Max. Precipitation | 0.003 |  |  | Min. Temperature*Average Temperature | 0.0004 |  |  |  |  |  |
|  |  | Duration*Year Precipitation | 0.0019 |  |  |  |  |  |  |  |  |  |
|  |  | Year Precipitation*Max. Precipitation | 0.004 |  |  |  |  |  |  |  |  |  |
|  |  | Year Precipitation*Min. Precipitation | <0.0001 |  |  |  |  |  |  |  |  |  |
| Ebullition | 78 | Estimation Method | <0.0001 | 0.85 | 46 | Area | 0.0099 | 0.98 | 19 | Max. Precipitation | 0.03 | 0.91 |
|  |  | Climatic Zone | <0.0007 |  |  | Landscape | <0.0001 |  |  | Average Temperature | 0.003 |  |
|  |  | Min. Temperature | 0.033 |  |  | Min. Temperature | 0.0022 |  |  | Year Precipitation*DOC | 0.02 |  |
|  |  | Average Temperature | 0.003 |  |  | Average Temperature | 0.0003 |  |  |  |  |  |
|  |  | Estimation Method*Max. Temperature | 0.039 |  |  | Area*  Landscape | <0.0001 |  |  |  |  |  |
|  |  | Estimation Method*Average Temperature | 0.024 |  |  |  |  |  |  |  |  |  |
|  |  | Climatic Zone*Max. Temperature | 0.010 |  |  |  |  |  |  |  |  |  |
|  |  | Climatic Zone*Average Temperature | 0.049 |  |  |  |  |  |  |  |  |  |
|  |  | Climatic Zone*Year Precipitation | 0.009 |  |  |  |  |  |  |  |  |  |
|  |  | Climatic Zone*Min. Precipitation | 0.022 |  |  |  |  |  |  |  |  |  |
|  |  | Min. Temperature*Average Temperature | 0.010 |  |  |  |  |  |  |  |  |  |
|  |  | Min. Temperature*Min. Precipitation | 0.044 |  |  |  |  |  |  |  |  |  |
|  |  | Max. Temperature*Average Temperature | 0.002 |  |  |  |  |  |  |  |  |  |
| Storage | 93 | Climatic Zone | 0.04 | 0.65 | 93 | Climatic Zone | 0.04 | 0.65 | .27 | Min. Temperature | 0.007 | 0.81 |
|  |  | Min. Temperature | <0.0001 |  |  | Min. Temperature | <0.0001 |  |  | Landscape | 0.046 |  |
|  |  | Max. Temperature | 0.02 |  |  | Max. Temperature | 0.02 |  |  |  |  |  |
|  |  | Year Precipitation | 0.0008 |  |  | Year Precipitation | 0.0008 |  |  |  |  |  |
|  |  | Min. Temperature*Min. Precipitation | <0.0001 |  |  | Min. Temperature*Min. Precipitation | <0.0001 |  |  |  |  |  |
|  |  | Starting Year*Duration | 0.0097 |  |  | Starting Year*Duration | 0.0097 |  |  |  |  |  |
|  |  | Starting year*Year Precipitation | 0.04 |  |  | Starting year*Year Precipitation | 0.04 |  |  |  |  |  |
|  |  | Duration*Min. Precipitation | 0.019 |  |  | Duration*Min. Precipitation | 0.019 |  |  |  |  |  |

Supporting Table 2: Source and extent of the lake data. The available data for each study are denoted by “x” in the appropriate columns. “Total” means all three types of open water fluxes i.e., diffusive (Diff), ebullitive (Ebul) and storage (Stor) flux. The numbers correspond to different variables: 1) Estimation component; 2) Estimation method; 3) Climatic zone; 4) Starting year of measurements; 5) Duration of the sampling event (in months); 6) Precipitation during the sampling year (annual mean in mm d^-1^); 7) Maximum monthly precipitation during the sampling year (monthly mean in mm d^-1^); 8) Minimum monthly precipitation during the sampling year (monthly mean in mm d^-1^); 9) Average air temperature during the sampling year (annual º C); 10) Maximum air temperature during the sampling year (monthly mean °C); 11) Minimum monthly air temperature during the sampling year (monthly mean °C); 12) Landscape; 13) Area; 14) Dissolved organic carbon (mg C l^-1^); 15) Maximal depth (m); 16) Phosphorus concentration (mmol l^-1^).

| **Climatic Zone** | **Source** | **Lake me** | Total | Diff | Ebul | Stor | 1,2,3 | **4** | **5** | **6** | **7** | **8** | **9** | **10** | **11** | **12** | **13** | **14** | **15** | **16** |
| --- | --- | --- | --- | --- | --- | --- | --- | --- | --- | --- | --- | --- | --- | --- | --- | --- | --- | --- | --- | --- |
| Boreal | Laurion et al. 2010 | BYL1 |  | x |  |  | x | x | x | x | x | x | x | x | x | x |  | x | x | x |
|  | Laurion et al. 2010 | BYL22 |  | x |  |  | x | x | x | x | x | x | x | x | x | x |  | x | x | x |
|  | Laurion et al. 2010 | BYL23 |  | x |  |  | x | x | x | x | x | x | x | x | x | x |  | x | x | x |
|  | Laurion et al. 2010 | BYL24 |  | x |  |  | x | x | x | x | x | x | x | x | x | x |  | x | x | x |
|  | Laurion et al. 2010 | BYL25 |  | x |  |  | x | x | x | x | x | x | x | x | x | x |  | x | x | x |
|  | Laurion et al. 2010 | BYL26 |  | x |  |  | x | x | x | x | x | x | x | x | x | x |  | x | x | x |
|  | Laurion et al. 2010 | BYL27 |  | x |  |  | x | x | x | x | x | x | x | x | x | x |  | x | x | x |
|  | Laurion et al. 2010 | BYL28 |  | x |  |  | x | x | x | x | x | x | x | x | x | x |  | x | x | x |
|  | Laurion et al. 2010 | BYL29 |  | x |  |  | x | x | x | x | x | x | x | x | x | x |  | x | x | x |
|  | Laurion et al. 2010 | BYL30 |  | x |  |  | x | x | x | x | x | x | x | x | x | x |  | x | x | x |
|  | Laurion et al. 2010 | BYL31 |  | x |  |  | x | x | x | x | x | x | x | x | x | x |  | x | x | x |
|  | Laurion et al. 2010 | BYL32 |  | x |  |  | x | x | x | x | x | x | x | x | x | x |  | x | x | x |
|  | Laurion et al. 2010 | BYL33 |  | x |  |  | x | x | x | x | x | x | x | x | x | x |  | x | x | x |
|  | Laurion et al. 2010 | BYL34 |  | x |  |  | x | x | x | x | x | x | x | x | x | x |  | x | x | x |
|  | Laurion et al. 2010 | BYL35 |  | x |  |  | x | x | x | x | x | x | x | x | x | x |  | x | x | x |
|  | Laurion et al. 2010 | BYL36 |  | x |  |  | x | x | x | x | x | x | x | x | x | x |  | x | x | x |
|  | Laurion et al. 2010 | BYL41 |  | x |  |  | x | x | x | x | x | x | x | x | x | x |  | x | x | x |
|  | Laurion et al. 2010 | BYL42 |  | x |  |  | x | x | x | x | x | x | x | x | x | x |  | x | x | x |
|  | Laurion et al. 2010 | BYL37 |  | x |  |  | x | x | x | x | x | x | x | x | x | x |  | x | x | x |
|  | Laurion et al. 2010 | BYL39 |  | x |  |  | x | x | x | x | x | x | x | x | x | x |  | x | x | x |
|  | Laurion et al. 2010 | BYL40 |  | x |  |  | x | x | x | x | x | x | x | x | x | x |  | x | x | x |
|  | Golubyatnikov and Kazantsev 2013 | Lake 1 - Gyda |  | x |  |  | x | x | x | x | x | x | x | x | x | x |  |  | x |  |
|  | Golubyatnikov and Kazantsev 2013 | Lake 2 - Gyda |  | x |  |  | x | x | x | x | x | x | x | x | x | x |  |  | x |  |
|  | Golubyatnikov and Kazantsev 2013 | Lake 3 - Gyda |  | x |  |  | x | x | x | x | x | x | x | x | x | x |  |  | x |  |
|  | Golubyatnikov and Kazantsev 2013 | Lake 4 - Gyda |  | x |  |  | x | x | x | x | x | x | x | x | x | x |  |  | x |  |
|  | Golubyatnikov and Kazantsev 2013 | Lake 5 - Gyda |  | x |  |  | x | x | x | x | x | x | x | x | x | x |  |  | x |  |
|  | Golubyatnikov and Kazantsev 2013 | Lake 1 - Yasavei |  | x |  |  | x | x | x | x | x | x | x | x | x | x |  |  | x |  |
|  | Golubyatnikov and Kazantsev 2013 | Lake 2 - Yasavei |  | x |  |  | x | x | x | x | x | x | x | x | x | x |  |  | x |  |
|  | Golubyatnikov and Kazantsev 2013 | Lake 3 - Yasavei |  | x |  |  | x | x | x | x | x | x | x | x | x | x |  |  | x |  |
|  | Golubyatnikov and Kazantsev 2013 | Lake 4 - Yasavei |  | x |  |  | x | x | x | x | x | x | x | x | x | x |  |  | x |  |
|  | Golubyatnikov and Kazantsev 2013 | Lake 5 - Yasavei |  | x |  |  | x | x | x | x | x | x | x | x | x | x |  |  | x |  |
|  | Golubyatnikov and Kazantsev 2013 | Lake 1 - Tazovskii |  | x |  |  | x | x | x | x | x | x | x | x | x | x |  |  | x |  |
|  | Golubyatnikov and Kazantsev 2013 | Lake 2 - Tazovskii |  | x |  |  | x | x | x | x | x | x | x | x | x | x |  |  | x |  |
|  | Golubyatnikov and Kazantsev 2013 | Lake 3 - Tazovskii |  | x |  |  | x | x | x | x | x | x | x | x | x | x |  |  | x |  |
|  | Golubyatnikov and Kazantsev 2013 | Lake 4 - Tazovskii |  | x |  |  | x | x | x | x | x | x | x | x | x | x |  |  | x |  |
|  | Golubyatnikov and Kazantsev 2013 | Lake 5 - Tazovskii |  | x |  |  | x | x | x | x | x | x | x | x | x | x |  |  | x |  |
|  | Golubyatnikov and Kazantsev 2013 | Lake 6 - Tazovskii |  | x |  |  | x | x | x | x | x | x | x | x | x | x |  |  | x |  |
|  | Walter et al. 2006 | Tube dispenser |  | x | x |  | x | x | x | x | x | x | x | x | x | x | x |  | x |  |
|  | Walter et al. 2006 | Shuchi |  | x | x |  | x | x | x | x | x | x | x | x | x | x | x |  | x |  |
|  | Kling et al. 1992 | Toolik |  | x |  |  | x | x | x | x | x | x | x | x | x | x | x | x | x |  |
|  | Kling et al. 1992 | N2 |  | x |  |  | x | x | x | x | x | x | x | x | x |  | x |  | x |  |
|  | Kling et al. 1992 | N1 |  | x |  |  | x | x | x | x | x | x | x | x | x |  | x |  | x | x |
|  | Kling et al. 1992 | Dam |  | x |  |  | x | x | x | x | x | x | x | x | x |  |  |  |  |  |
|  | Kling et al. 1992 | William |  | x |  |  | x | x | x | x | x | x | x | x | x |  |  |  |  |  |
|  | Kling et al. 1992 | Coleen |  | x |  |  | x | x | x | x | x | x | x | x | x |  |  |  |  |  |
|  | Kling et al. 1992 | Windy |  | x |  |  | x | x | x | x | x | x | x | x | x |  |  |  |  |  |
|  | Kling et al. 1992 | Pond 386 |  | x |  |  | x | x | x | x | x | x | x | x | x |  |  |  |  |  |
|  | Walter Anthony et al. 2010 | Tube Dispencer |  | x |  | x | x | x | x | x | x | x | x | x | x | x | x | x | x |  |
|  | Walter Anthony et al. 2010 | Shuchi |  | x |  | x | x | x | x | x | x | x | x | x | x | x | x | x | x |  |
|  | Walter Anthony et al. 2010 | Grass |  | x |  | x | x | x | x | x | x | x | x | x | x | x | x | x | x |  |
|  | Karlsson et al. 2013 | 1 |  | x |  | x | x | x | x | x | x | x | x | x | x | x | x |  | x |  |
|  | Karlsson et al. 2013 | 2 |  | x |  | x | x | x | x | x | x | x | x | x | x | x | x |  | x |  |
|  | Karlsson et al. 2013 | 3 |  | x |  | x | x | x | x | x | x | x | x | x | x | x | x |  | x |  |
|  | Karlsson et al. 2013 | 4 |  | x |  | x | x | x | x | x | x | x | x | x | x | x | x |  | x |  |
|  | Karlsson et al. 2013 | 6 |  | x |  | x | x | x | x | x | x | x | x | x | x | x | x |  | x |  |
|  | Karlsson et al. 2013 | 7 |  | x |  | x | x | x | x | x | x | x | x | x | x | x | x |  | x |  |
|  | Karlsson et al. 2013 | 8 |  | x |  | x | x | x | x | x | x | x | x | x | x | x | x |  | x |  |
|  | Karlsson et al. 2013 | 9 |  | x |  | x | x | x | x | x | x | x | x | x | x | x | x |  | x |  |
|  | Karlsson et al. 2013 | 13 |  | x |  | x | x | x | x | x | x | x | x | x | x | x | x |  | x |  |
|  | Karlsson et al. 2013 | 14 |  | x |  | x | x | x | x | x | x | x | x | x | x | x | x |  | x |  |
|  | Karlsson et al. 2013 | 20 |  | x |  | x | x | x | x | x | x | x | x | x | x | x | x |  | x |  |
|  | Karlsson et al. 2013 | 24 |  | x |  | x | x | x | x | x | x | x | x | x | x | x | x |  | x |  |
|  | Wik et al. 2013 | Inre Harrsjön |  |  | x |  | x | x | x | x | x | x | x | x | x | x | x |  | x |  |
|  | Wik et al. 2013 | Mellan Harrsjön |  |  | x |  | x | x | x | x | x | x | x | x | x | x | x |  | x |  |
|  | Wik et al. 2013 | Villasjön |  |  | x |  | x | x | x | x | x | x | x | x | x | x | x |  | x |  |
|  | Thompson et al. 2017 | EVV Upper lake |  | x | x |  | x | x | x | x | x | x | x | x | x | x | x | x | x |  |
|  | Sepulveda-Jauregui et al. 2015 | Big Sky | x | x | x | x | x | x | x | x | x | x | x | x | x | x | x | x | x | x |
|  | Sepulveda-Jauregui et al. 2015 | Dragon’s Pond |  | x |  | x | x | x | x | x | x | x | x | x | x | x | x | x | x | x |
|  | Sepulveda-Jauregui et al. 2015 | GTH 112 |  |  | x | x | x | x | x | x | x | x | x | x | x | x | x |  | x |  |
|  | Sepulveda-Jauregui et al. 2015 | NE2 | x | x | x | x | x | x | x | x | x | x | x | x | x | x | x |  | x | x |
|  | Sepulveda-Jauregui et al. 2015 | E6 |  | x |  | x | x | x | x | x | x | x | x | x | x | x | x |  | x | x |
|  | Sepulveda-Jauregui et al. 2015 | E5 Oil Spill A30 |  | x |  | x | x | x | x | x | x | x | x | x | x | x | x |  | x | x |
|  | Sepulveda-Jauregui et al. 2015 | Toolik A28 | x | x | x | x | x | x | x | x | x | x | x | x | x | x | x | x | x | x |
|  | Sepulveda-Jauregui et al. 2015 | E1 | x | x | x | x | x | x | x | x | x | x | x | x | x | x | x |  | x | x |
|  | Sepulveda-Jauregui et al. 2015 | Autumn |  | x |  | x | x | x | x | x | x | x | x | x | x | x | x | x | x | x |
|  | Sepulveda-Jauregui et al. 2015 | Julieta | x | x | x | x | x | x | x | x | x | x | x | x | x | x | x | x | x | x |
|  | Sepulveda-Jauregui et al. 2015 | El Fuego |  | x |  |  | x | x | x | x | x | x | x | x | x | x | x |  | x |  |
|  | Sepulveda-Jauregui et al. 2015 | Jos |  | x | x |  | x | x | x | x | x | x | x | x | x | x | x | x | x | x |
|  | Sepulveda-Jauregui et al. 2015 | Augustine Zoli |  | x |  | x | x | x | x | x | x | x | x | x | x | x | x | x | x | x |
|  | Sepulveda-Jauregui et al. 2015 | Ping | x | x | x | x | x | x | x | x | x | x | x | x | x | x | x | x | x |  |
| North temperate | Sepulveda-Jauregui et al. 2015 | Grayling | x | x | x | x | x | x | x | x | x | x | x | x | x | x | x | x | x | x |
|  | Sepulveda-Jauregui et al. 2015 | Eugenia |  |  | x | x | x | x | x | x | x | x | x | x | x | x | x | x | x |  |
|  | Sepulveda-Jauregui et al. 2015 | Vault |  | x |  | x | x | x | x | x | x | x | x | x | x | x | x |  | x |  |
|  | Sepulveda-Jauregui et al. 2015 | Goldstream | x | x | x | x | x | x | x | x | x | x | x | x | x | x | x |  | x | x |
|  | Sepulveda-Jauregui et al. 2015 | Doughnut |  |  |  | x | x | x | x | x | x | x | x | x | x | x | x |  | x |  |
|  | Sepulveda-Jauregui et al. 2015 | Killarney |  | x |  | x | x | x | x | x | x | x | x | x | x | x | x | x | x | x |
|  | Sepulveda-Jauregui et al. 2015 | Smith | x | x | x | x | x | x | x | x | x | x | x | x | x | x | x |  | x | x |
|  | Sepulveda-Jauregui et al. 2015 | Stevens Pond |  | x |  | x | x | x | x | x | x | x | x | x | x | x | x |  | x |  |
|  | Sepulveda-Jauregui et al. 2015 | Duece |  | x |  |  | x | x | x | x | x | x | x | x | x | x | x |  | x | x |
|  | Sepulveda-Jauregui et al. 2015 | Ace |  | x |  |  | x | x | x | x | x | x | x | x | x | x | x |  | x | x |
|  | Sepulveda-Jauregui et al. 2015 | Rosie Creek | x | x | x | x | x | x | x | x | x | x | x | x | x | x | x |  | x |  |
|  | Sepulveda-Jauregui et al. 2015 | Mosta |  | x |  |  | x | x | x | x | x | x | x | x | x | x | x | x | x | x |
|  | Sepulveda-Jauregui et al. 2015 | 91 Lake |  | x |  | x | x | x | x | x | x | x | x | x | x | x | x |  | x |  |
|  | Sepulveda-Jauregui et al. 2015 | Otto | x | x | x | x | x | x | x | x | x | x | x | x | x | x | x | x | x | x |
|  | Sepulveda-Jauregui et al. 2015 | Floatplane |  |  |  | x | x | x | x | x | x | x | x | x | x | x | x |  | x | x |
|  | Sepulveda-Jauregui et al. 2015 | Nutella |  | x |  | x | x | x | x | x | x | x | x | x | x | x | x |  | x | x |
|  | Sepulveda-Jauregui et al. 2015 | Swampbuggy |  | x |  | x | x | x | x | x | x | x | x | x | x | x | x |  | x | x |
|  | Sepulveda-Jauregui et al. 2015 | Monta | x | x | x | x | x | x | x | x | x | x | x | x | x | x | x | x | x | x |
|  | Sepulveda-Jauregui et al. 2015 | Rainbow Shore |  | x | x |  | x | x | x | x | x | x | x | x | x | x | x | x | x | x |
|  | Sepulveda-Jauregui et al. 2015 | Big Merganser | x | x | x | x | x | x | x | x | x | x | x | x | x | x | x | x | x | x |
|  | Sepulveda-Jauregui et al. 2015 | Rainbow |  | x | x |  | x | x | x | x | x | x | x | x | x | x | x | x | x | x |
|  | Sepulveda-Jauregui et al. 2015 | Dolly Varden | x | x | x | x | x | x | x | x | x | x | x | x | x | x | x |  | x | x |
|  | Sepulveda-Jauregui et al. 2015 | Abandoned Cabin |  | x |  |  | x | x | x | x | x | x | x | x | x | x | x |  | x | x |
|  | Sepulveda-Jauregui et al. 2015 | Scout |  |  | x | x | x | x | x | x | x | x | x | x | x | x | x | x | x | x |
|  | Sepulveda-Jauregui et al. 2015 | Engineer | x | x | x | x | x | x | x | x | x | x | x | x | x | x | x | x | x | x |
|  | Sepulveda-Jauregui et al. 2015 | Lower Ohmer |  | x |  | x | x | x | x | x | x | x | x | x | x | x | x |  | x | x |
|  | Repo et al. 2007 | Siberian tundra lake |  | x | x |  | x | x | x | x | x | x | x | x | x | x | x |  |  | x |
|  | Bastviken et al. 2002 | Illersjön |  | x |  | x | x | x | x | x | x | x | x | x | x | x | x | x | x | x |
|  | Bastviken et al. 2002 | Mårn |  | x |  | x | x | x | x | x | x | x | x | x | x | x | x | x | x | x |
|  | Bastviken et al. 2002 | Lillsjön |  | x |  | x | x | x | x | x | x | x | x | x | x | x | x | x | x | x |
|  | Huttunen et al. 2003 | Postilampi | x | x | x | x | x | x | x | x | x | x | x | x | x | x | x |  | x |  |
|  | Huttunen et al. 2003 | Kevätön | x | x | x | x | x | x | x | x | x | x | x | x | x | x | x |  | x |  |
|  | Huttunen et al. 2003 | Vehmasjärvi |  | x |  |  | x | x | x | x | x | x | x | x | x | x | x |  | x |  |
|  | Huttunen et al. 2003 | Mäkijärvi |  | x |  |  | x | x | x | x | x | x | x | x | x | x | x |  | x |  |
|  | Juutinen et al. 2003 | Kevatön |  | x |  |  | x | x | x | x | x | x | x | x | x | x | x |  | x |  |
|  | Juutinen et al. 2003 | Heposälkä |  | x |  |  | x | x | x | x | x | x | x | x | x | x | x |  | x |  |
|  | Juutinen et al. 2003 | Mekrijärvi |  | x |  |  | x | x | x | x | x | x | x | x | x | x | x |  | x |  |
|  | Kankaala et al. 2006 | Valkea-Kotinen |  | x |  |  | x | x | x | x | x | x | x | x | x | x | x | x | x | x |
|  | Phelps et al. 1998 | Goose lake |  | x |  |  | x | x | x | x | x | x | x | x | x | x | x |  | x |  |
|  | Phelps et al 1998 | Mosquito |  |  |  | x | x | x | x | x | x | x | x | x | x |  |  |  |  |  |
|  | Ojala et al 2011 | Lake Paajarvi |  | x |  |  | x | x | x | x | x | x | x | x | x | x | x | x | x |  |
|  | Ojala et al 2011 | Lake Paajarvi |  | x |  |  | x | x | x | x | x | x | x | x | x | x | x | x | x |  |
|  | Ojala et al 2011 | Lake Ormajavi |  | x |  |  | x | x | x | x | x | x | x | x | x | x | x | x | x |  |
|  | Ojala et al 2011 | Lake Ormajavi |  | x |  |  | x | x | x | x | x | x | x | x | x | x | x | x | x |  |
|  | Repo et al. 2007 | Siberian taiga lake |  | x | x |  | x | x | x | x | x | x | x | x | x | x | x |  |  | x |
|  | Repo et al. 2007 | Siberian taiga pond |  | x |  |  | x | x | x | x | x | x | x | x | x | x | x |  |  | x |
|  | Bartlett et al 1992 | Pingo pond |  | x |  |  | x | x | x | x | x | x | x | x | x | x |  |  | x |  |
|  | Sabrekov et al. 2017 | Bondarevskoe |  | x | x |  | x | x | x | x | x | x | x | x | x | x | x | x | x | x |
|  | Sabrekov et al. 2017 | Lebedinoe |  | x |  |  | x | x | x | x | x | x | x | x | x | x | x | x | x | x |
|  | Sabrekov et al. 2017 | Babochka |  | x |  |  | x | x | x | x | x | x | x | x | x | x | x | x | x | x |
|  | Sabrekov et al. 2017 | Muhrino |  | x | x |  | x | x | x | x | x | x | x | x | x | x | x | x | x | x |
|  | Sabrekov et al. 2017 | Bakchar-ryam |  | x |  |  | x | x | x | x | x | x | x | x | x | x | x | x | x | x |
|  | Sabrekov et al. 2017 | Bakchar-forest-1 |  | x | x |  | x | x | x | x | x | x | x | x | x | x | x | x | x | x |
|  | Sabrekov et al. 2017 | Bakchar-forest-2 |  | x |  |  | x | x | x | x | x | x | x | x | x | x | x | x | x | x |
|  | Sabrekov et al. 2017 | Bakchar-forest-3 |  | x | x |  | x | x | x | x | x | x | x | x | x | x | x | x | x | x |
|  | Sabrekov et al. 2017 | Gavrilovka-1 |  | x |  |  | x | x | x | x | x | x | x | x | x | x | x | x | x | x |
|  | Sabrekov et al. 2017 | Gavrilovka-2 |  | x |  |  | x | x | x | x | x | x | x | x | x | x | x | x | x | x |
|  | Sabrekov et al. 2017 | Bakchar-bog-1 |  | x | x |  | x | x | x | x | x | x | x | x | x | x | x | x | x | x |
|  | Sabrekov et al. 2017 | Bakchar-bog-2 |  | x |  |  | x | x | x | x | x | x | x | x | x | x | x | x | x | x |
|  | Sabrekov et al. 2017 | Plotnikovo |  | x |  |  | x | x | x | x | x | x | x | x | x | x | x | x | x | x |
|  | Sabrekov et al. 2017 | Ob’ Floodplain |  | x |  |  | x | x | x | x | x | x | x | x | x | x | x | x | x | x |
|  | Natchimuthu et al 2014 | small shallow pond |  | x | x |  | x | x | x | x | x | x | x | x | x | x | x |  | x |  |
|  | Natchimuthu et al 2016 | Erssjon |  | x |  |  | x | x | x | x | x | x | x | x | x | x | x |  | x |  |
|  | Natchimuthu et al 2016 | Foljesjon |  | x |  |  | x | x | x | x | x | x | x | x | x | x | x |  | x |  |
|  | Bastviken et al. 2004 | Bisen |  | x |  |  | x | x | x | x | x | x | x | x | x | x | x | x |  | x |
|  | Bastviken et al. 2004 | L Sangaren |  | x |  | x | x | x | x | x | x | x | x | x | x | x | x | x |  | x |
|  | Bastviken et al. 2004 | Ljustjarn |  | x |  | x | x | x | x | x | x | x | x | x | x | x | x | x |  | x |
|  | Bastviken et al. 2004 | Lovtjarn |  | x |  |  | x | x | x |  |  |  |  |  |  | x | x | x |  | x |
|  | Bastviken et al. 2004 | Ragastjarn |  | x |  |  | x | x | x | x | x | x | x | x | x | x | x | x |  | x |
|  | Bastviken et al. 2004 | Skottjarn |  | x |  |  | x | x | x |  |  |  |  |  |  |  | x | x |  | x |
|  | Bastviken et al. 2004 | Svarttjarn |  | x |  |  | x | x | x | x | x | x | x | x | x | x | x | x |  | x |
|  | Bartlett et al 1992 | Chaos |  | x |  |  | x | x | x | x | x | x | x | x | x |  |  |  | x |  |
|  | Bastviken et al. 2004 | Fiolen |  | x |  | x | x | x | x | x | x | x | x | x | x | x | x | x |  | x |
|  | Bastviken et al. 2004 | Gransjon |  | x |  |  | x | x | x | x | x | x | x | x | x | x | x | x |  | x |
|  | Bastviken et al. 2004 | Grunnen |  | x |  |  | x | x | x | x | x | x | x | x | x | x | x | x |  | x |
|  | Bastviken et al. 2004 | Gyslattasjon |  | x |  |  | x | x | x | x | x | x | x | x | x | x | x | x |  | x |
|  | Bastviken et al. 2004 | Klintsjon |  | x |  | x | x | x | x | x | x | x | x | x | x | x | x | x |  | x |
|  | Bastviken et al. 2004 | Skarshultssjon |  | x |  | x | x | x | x | x | x | x | x | x | x | x | x | x |  | x |
|  | Laurion et al. 2010 | KWK1 |  | x |  |  | x | x | x | x | x | x | x | x | x | x |  | x | x | x |
|  | Laurion et al. 2010 | KWK2 |  | x |  |  | x | x | x | x | x | x | x | x | x | x |  | x | x | x |
|  | Laurion et al. 2010 | KWK3 |  | x |  |  | x | x | x | x | x | x | x | x | x | x |  | x | x | x |
|  | Laurion et al. 2010 | KWK6 |  | x |  |  | x | x | x | x | x | x | x | x | x | x |  | x | x | x |
|  | Laurion et al. 2010 | KWK7 |  | x |  |  | x | x | x | x | x | x | x | x | x | x |  | x | x | x |
|  | Laurion et al. 2010 | KWK11 |  | x |  |  | x | x | x | x | x | x | x | x | x | x |  | x | x | x |
|  | Laurion et al. 2010 | KWK21 |  | x |  |  | x | x | x | x | x | x | x | x | x | x |  | x | x | x |
|  | Laurion et al. 2010 | KWK23 |  | x |  |  | x | x | x | x | x | x | x | x | x | x |  | x | x | x |
|  | Laurion et al. 2010 | KWK33 |  | x |  |  | x | x | x | x | x | x | x | x | x | x |  | x | x | x |
|  | Laurion et al. 2010 | KWK35 |  | x |  |  | x | x | x | x | x | x | x | x | x | x |  | x | x | x |
|  | Laurion et al. 2010 | KWK36 |  | x |  |  | x | x | x | x | x | x | x | x | x | x |  | x | x | x |
|  | Laurion et al. 2010 | KWK38 |  | x |  |  | x | x | x | x | x | x | x | x | x | x |  | x | x | x |
|  | Casper et al. 2000 | Priest Pot | x | x | x | x | x | x | x | x | x | x | x | x | x | x | x |  | x | x |
|  | Schrier-Uijl et al. 2011 | Reeuwijkse plas |  | x |  |  | x | x | x | x | x | x | x | x | x | x | x |  |  | x |
|  | Schrier-Uijl et al. 2011 | Vinkeveense plas |  | x |  |  | x | x | x | x | x | x | x | x | x | x | x |  |  | x |
|  | Schrier-Uijl et al. 2011 | Nieuwkoopse plas |  | x |  |  | x | x | x | x | x | x | x | x | x | x | x |  |  | x |
|  | Schrier-Uijl et al. 2011 | Belterwiede |  | x |  |  | x | x | x | x | x | x | x | x | x | x | x |  |  | x |
|  | Schrier-Uijl et al. 2011 | Schutsloterwiede |  | x |  |  | x | x | x | x | x | x | x | x | x | x | x |  |  | x |
|  | Demarty et al 2011 | Mistumis Lake |  | x |  |  | x | x | x | x | x | x | x | x | x | x | x |  | x |  |
|  | Demarty et al 2011 | Clarkie Lake |  | x |  |  | x | x | x | x | x | x | x | x | x | x | x |  | x |  |
|  | Rudd & Hamilton 1978 | Lake 227 |  | x |  | x | x |  |  |  |  |  |  |  |  | x | x | x |  | x |
|  | Pelletier et al. 2014 | 1 |  | x |  |  | x | x | x | x | x | x | x | x | x | x | x | x | x |  |
|  | Pelletier et al. 2014 | 2 |  | x |  |  | x | x | x | x | x | x | x | x | x | x | x | x | x |  |
|  | Pelletier et al. 2014 | 3 |  | x |  |  | x | x | x | x | x | x | x | x | x | x | x | x | x |  |
|  | Pelletier et al. 2014 | 4 |  | x |  |  | x | x | x | x | x | x | x | x | x | x | x | x | x |  |
|  | Pelletier et al. 2014 | 5 |  | x |  |  | x | x | x | x | x | x | x | x | x | x | x | x | x |  |
|  | Tangen et al. 2016 | Long Lake |  | x |  |  | x | x | x | x | x | x | x | x | x | x | x |  | x |  |
|  | Striegl & Michmerhuizen 1998 | Williams |  |  |  | x | x | x | x | x | x | x | x | x | x | x | x |  | x |  |
|  | Striegl & Michmerhuizen 1998 | Shingboee |  |  |  | x | x | x | x | x | x | x | x | x | x | x | x |  | x |  |
|  | Michmerhuizen et al. 1996 | Little Shingboee |  |  |  | x | x | x | x | x | x | x | x | x | x | x | x |  | x |  |
|  | Michmerhuizen et al. 1996 | 11th Crow Wing |  |  |  | x | x | x | x | x | x | x | x | x | x | x | x |  | x |  |
|  | Michmerhuizen et al. 1996 | Leech |  |  |  | x | x | x | x | x | x | x | x | x | x | x | x |  | x |  |
|  | Michmerhuizen et al. 1996 | Allequash |  |  |  | x | x | x | x | x | x | x | x | x | x | x | x | x | x | x |
|  | Michmerhuizen et al. 1996 | Big Muskellunge |  |  |  | x | x | x | x | x | x | x | x | x | x | x | x | x | x | x |
|  | Michmerhuizen et al. 1996 | Trout |  |  |  | x | x | x | x | x | x | x | x | x | x | x | x | x | x | x |
|  | Michmerhuizen et al. 1996 | Glacier pond |  |  |  | x | x | x | x | x | x | x | x | x | x | x | x |  | x |  |
|  | Michmerhuizen et al. 1996 | Tofte |  |  |  | x | x | x | x | x | x | x | x | x | x | x | x |  | x |  |
|  | Michmerhuizen et al. 1996 | Jasper |  |  |  | x | x | x | x | x | x | x | x | x | x | x | x | x | x |  |
|  | Michmerhuizen et al. 1996 | Ojibway |  |  |  | x | x | x | x | x | x | x | x | x | x | x | x |  | x |  |
|  | Michmerhuizen et al. 1996 | Snowbank |  |  |  | x | x | x | x | x | x | x | x | x | x | x | x | x | x |  |
|  | Michmerhuizen et al. 1996 | Hiawatha |  |  |  | x | x | x | x | x | x | x | x | x | x | x | x | x | x |  |
|  | Michmerhuizen et al. 1996 | Nokomis |  |  |  | x | x | x | x | x | x | x | x | x | x | x | x | x | x |  |
|  | Michmerhuizen et al. 1996 | Harriet |  |  |  | x | x | x | x | x | x | x | x | x | x | x | x | x | x |  |
|  | Michmerhuizen et al. 1996 | Calhoun |  |  |  | x | x | x | x | x | x | x | x | x | x | x | x |  | x |  |
|  | Michmerhuizen et al. 1996 | Minnetonka |  |  |  | x | x | x | x | x | x | x | x | x | x | x | x |  | x |  |
|  | Michmerhuizen et al. 1996;Riera et al. 1999 | Crystal |  | x |  | x | x | x | x | x | x | x | x | x | x | x | x | x | x |  |
|  | Riera et al. 1999 | Crystal Bog |  | x |  | x | x | x | x | x | x | x | x | x | x | x | x | x | x |  |
|  | Riera et al. 1999 | Trout Bog |  | x |  | x | x | x | x | x | x | x | x | x | x | x | x | x | x |  |
|  | Riera et al. 1999 | Sparkling |  | x |  | x | x | x | x | x | x | x | x | x | x | x | x | x | x |  |
|  | Chau et al 1977 | Ontario |  |  | x |  | x |  |  |  |  |  |  |  |  |  | x | x | x | x |
|  | Bastviken et al. 2004 | Brown | x | x | x | x | x | x | x | x | x | x | x | x | x | x | x | x | x | x |
|  | Bastviken et al. 2004 | Crampton | x | x | x | x | x | x | x | x | x | x | x | x | x | x | x | x | x | x |
|  | Bastviken et al. 2004 | East Long | x | x | x | x | x | x | x | x | x | x | x | x | x | x | x | x | x | x |
|  | Bastviken et al. 2004 | Hummingbird | x | x | x | x | x | x | x | x | x | x | x | x | x | x | x | x |  | x |
|  | Bastviken et al. 2004 | Morris | x | x | x | x | x | x | x | x | x | x | x | x | x | x | x | x | x | x |
|  | Bastviken et al. 2004 | North Gate | x | x | x | x | x | x | x | x | x | x | x | x | x | x | x | x |  | x |
|  | Bastviken et al. 2004 | Paul | x | x | x | x | x | x | x | x | x | x | x | x | x | x | x | x | x | x |
|  | Bastviken et al. 2004 | Peter | x | x | x | x | x | x | x | x | x | x | x | x | x | x | x | x |  | x |
|  | Bastviken et al. 2004 | Roach | x | x | x | x | x | x | x | x | x | x | x | x | x | x | x | x | x | x |
|  | Bastviken et al. 2004 | Tuesday | x | x | x | x | x | x | x | x | x | x | x | x | x | x | x | x |  | x |
|  | Bastviken et al. 2004 | Ward | x | x | x | x | x | x | x | x | x | x | x | x | x | x | x | x | x | x |
|  | Matthews et al. 2005 | Onodaga |  |  | x |  | x | x | x | x | x | x | x | x | x | x | x |  | x |  |
|  | Fallon et al. 1980 | Lake Mendota |  | x |  | x | x | x | x | x | x | x | x | x | x | x | x | x | x | x |
|  | Strayer & Tiedje 1978 | Wintergreen |  | x | x |  | x | x | x | x | x | x | x | x | x | x | x | x | x |  |
|  | West et al 2015 | Bay |  | x |  |  | x | x | x | x | x | x | x | x | x | x | x | x | x | x |
|  | West et al 2015 | Bergner |  | x |  |  | x | x | x | x | x | x | x | x | x | x | x | x | x | x |
|  | West et al 2015 | Brown |  | x |  |  | x | x | x | x | x | x | x | x | x | x | x | x | x | x |
|  | West et al 2015 | Cranberry |  | x |  |  | x | x | x | x | x | x | x | x | x | x | x | x | x | x |
|  | West et al 2015 | Crampton |  | x |  |  | x | x | x | x | x | x | x | x | x | x | x | x | x | x |
|  | West et al 2015 | Foggy |  | x |  |  | x | x | x | x | x | x | x | x | x | x | x | x | x | x |
|  | West et al 2015 | Hummingbird |  | x |  |  | x | x | x | x | x | x | x | x | x | x | x | x | x | x |
|  | West et al 2015 | Misty |  | x |  |  | x | x | x | x | x | x | x | x | x | x | x | x | x | x |
|  | West et al 2015 | Morris |  | x |  |  | x | x | x | x | x | x | x | x | x | x | x | x | x | x |
|  | West et al 2015 | North Gate |  | x |  |  | x | x | x | x | x | x | x | x | x | x | x | x | x | x |
|  | West et al 2015 | Paul |  | x |  |  | x | x | x | x | x | x | x | x | x | x | x | x | x | x |
|  | West et al 2015 | Peter |  | x |  |  | x | x | x | x | x | x | x | x | x | x | x | x | x | x |
|  | West et al 2015 | Raspberry |  | x |  |  | x | x | x | x | x | x | x | x | x | x | x | x | x | x |
|  | West et al 2015 | Tuesday |  | x |  |  | x | x | x | x | x | x | x | x | x | x | x | x | x | x |
|  | West et al 2015 | West Long |  | x |  |  | x | x | x | x | x | x | x | x | x | x | x | x | x | x |
|  | Smith & Lewis 1992 | Dillon |  | x |  |  | x | x | x | x | x | x | x | x | x | x | x |  | x |  |
|  | Smith & Lewis 1992 | Red Rock |  | x |  |  | x | x | x | x | x | x | x | x | x | x | x | x | x |  |
|  | Smith & Lewis 1992 | Rainbow |  | x |  |  | x | x | x | x | x | x | x | x | x | x | x | x | x |  |
|  | Smith & Lewis 1992 | Long |  | x |  |  | x | x | x | x | x | x | x | x | x |  | x |  | x |  |
|  | Smith & Lewis 1992 | Pass |  | x |  |  | x | x | x | x | x | x | x | x | x | x | x |  | x |  |
|  | Mattson & Likens 1990 | Mirror |  |  | x |  | x | x | x | x | x | x | x | x | x | x | x | x | x |  |
|  | Miller & Oremland 1988 | Big Soda |  | x |  |  | x | x | x | x | x | x | x | x | x | x | x |  |  |  |
|  | Miller & Oremland 1988 | Soap |  | x |  |  | x | x | x | x | x | x | x | x | x | x | x |  | x |  |
|  | Miller & Oremland 1988 | Mono |  | x |  |  | x | x | x | x | x | x | x | x | x | x | x |  | x |  |
|  | Miller & Oremland 1988 | Searsville |  | x |  |  | x | x | x | x | x | x | x | x | x | x | x |  |  |  |
|  | Utsuumi et al. 1998 a | Kasumigaura |  | x |  |  | x | x | x | x | x | x | x | x | x | x | x |  | x | x |
|  | Utsuumi et al. 1998 b | Nojiri |  | x |  |  | x | x | x | x | x | x | x | x | x | x | x |  | x |  |
|  | Miyajima et al. 1997 | Biwa |  | x |  |  | x | x | x | x | x | x | x | x | x | x | x |  | x | x |
|  | Xing et al. 2005 | Donghu |  | x |  |  | x | x | x | x | x | x | x | x | x | x | x | x | x |  |
| Tropical | Attermeyer et al. 2016 | Thimmapuram |  | x | x |  | x | x | x | x | x | x | x | x | x | x | x |  |  |  |
|  | Selvam et al 2014 | SUKA P1 |  |  | x |  | x | x | x | x | x | x | x | x | x | x |  |  |  |  |
|  | Selvam et al 2014 | Puliyalam Lake (SUPU L1) |  | x | x |  | x | x | x | x | x | x | x | x | x | x |  |  |  |  |
|  | Selvam et al 2014 | Nemmeli Lake (CNNE L1) |  | x | x |  | x | x | x | x | x | x | x | x | x | x |  |  |  |  |
|  | Selvam et al 2014 | Madhurandhagam Lake (MAKA L1) |  | x | x |  | x | x | x | x | x | x | x | x | x | x |  |  |  |  |
|  | Selvam et al 2014 | Kolavai Lake (CNKO L1) |  | x | x |  | x | x | x | x | x | x | x | x | x | x |  |  |  |  |
|  | Selvam et al 2014 | DHPE P1 Pond |  | x | x |  | x | x | x | x | x | x | x | x | x | x |  |  |  |  |
|  | Selvam et al 2014 | DHKO L1 Lake |  | x | x |  | x | x | x | x | x | x | x | x | x | x |  |  |  |  |
|  | Selvam et al 2014 | CPST P1 Pond |  | x | x |  | x | x | x | x | x | x | x | x | x | x |  |  |  |  |
|  | Selvam et al 2014 | CTRT P1 Pond |  | x | x |  | x | x | x | x | x | x | x | x | x | x |  |  |  |  |
|  | Selvam et al 2014 | KAMK Pond |  | x | x |  | x | x | x | x | x | x | x | x | x | x |  |  |  |  |
|  | Selvam et al 2014 | TSFP1Pond |  | x |  |  | x | x | x | x | x | x | x | x | x | x |  |  |  |  |
|  | Selvam et al 2014 | THKP1 Pond |  |  | x |  | x | x | x | x | x | x | x | x | x | x |  |  |  |  |
|  | Selvam et al 2014 | TSFP2 Pond |  | x | x |  | x | x | x | x | x | x | x | x | x | x |  |  |  |  |
|  | Selvam et al 2014 | Parakkai Lake (NPSK) |  |  | x |  | x | x | x | x | x | x | x | x | x | x |  |  |  |  |
|  | Smith et al. 2000 | Orinico river flodplain |  | x | x |  | x | x | x | x | x | x | x | x | x | x |  |  |  |  |
|  | Engle and Melack 2000, Crill et al 1988 | Calado | x | x | x | x | x | x | x | x | x | x | x | x | x | x | x |  | x |  |
|  | Peixoto et al. 2015 | floodplain lake |  | x |  |  | x | x | x | x | x | x | x | x | x | x |  |  | x |  |
|  | Marani & Alvalá 2007 | Mirante |  | x | x |  | x | x | x | x | x | x | x | x | x | x |  |  | x |  |
|  | Marani & Alvalá 2007 | Medalha |  | x | x |  | x | x | x | x | x | x | x | x | x | x |  |  |  |  |
|  | Bartlett et al. 1988 | Cabalia |  | x | x |  | x | x | x | x | x | x | x | x | x |  |  |  |  |  |
|  | Bastviken et al. 2010 | Belém |  | x | x |  | x | x | x | x | x | x | x | x | x | x |  |  |  |  |
|  | Bastviken et al. 2010 | Presa |  | x | x |  | x | x | x | x | x | x | x | x | x | x |  |  |  |  |
|  | Bastviken et al. 2010 | Bracinho |  | x | x |  | x | x | x | x | x | x | x | x | x | x |  |  |  |  |
|  | Bastviken et al. 2010 | Lobo |  | x | x |  | x | x | x | x | x | x | x | x | x | x |  |  |  |  |
|  | Bastviken et al. 2010 | Tereza |  | x | x |  | x | x | x | x | x | x | x | x | x | x |  |  |  |  |
|  | Bastviken et al. 2010 | L 1 |  | x | x |  | x | x | x | x | x | x | x | x | x | x |  |  |  |  |
|  | Bastviken et al. 2010 | L 2 |  | x | x |  | x | x | x | x | x | x | x | x | x | x |  |  |  |  |
|  | Bastviken et al. 2010 | L 3 |  | x | x |  | x | x | x | x | x | x | x | x | x | x |  |  |  |  |
|  | Bastviken et al. 2010 | L 4 |  | x | x |  | x | x | x | x | x | x | x | x | x | x |  |  |  |  |
|  | Bastviken et al. 2010 | TR |  | x | x |  | x | x | x | x | x | x | x | x | x | x |  |  |  |  |
|  | Bastviken et al. 2010 | BB |  | x | x |  | x | x | x | x | x | x | x | x | x | x |  |  |  |  |
|  | Bastviken et al. 2010 | N6b |  | x | x |  | x | x | x | x | x | x | x | x | x | x |  |  |  |  |
|  | Bastviken et al. 2010 | N7a |  | x | x |  | x | x | x | x | x | x | x | x | x | x |  |  |  |  |
|  | Bastviken et al. 2010 | N8a |  | x | x |  | x | x | x | x | x | x | x | x | x | x |  |  |  |  |
|  | Bastviken et al. 2010 | N14 |  | x | x |  | x | x | x | x | x | x | x | x | x | x |  |  |  |  |
|  | Bastviken et al. 2010 | n19a |  | x | x |  | x | x | x | x | x | x | x | x | x | x |  |  |  |  |
|  | Marinho 2013 | Imboassica |  | x |  |  | x | x | x | x | x | x | x | x | x | x | x | x | x |  |
|  | Marinho 2013 | Cabius |  | x |  |  | x | x | x | x | x | x | x | x | x | x | x | x | x |  |
|  | Marinho 2013 | Comprida |  | x |  |  | x | x | x | x | x | x | x | x | x | x | x | x | x |  |
|  | Marinho 2013 | Carapebus |  | x |  |  | x | x | x | x | x | x | x | x | x | x | x | x | x |  |
|  | Marinho 2013 | PiriPiri |  | x |  |  | x | x | x | x | x | x | x | x | x | x | x | x | x |  |
| South temperate | Palma-Silva et. al. 2013 | Polegar |  | x |  |  | x | x | x | x | x | x | x | x | x | x | x |  | x |  |
|  | Palma-Silva et. al. 2013 | Biguás |  | x |  |  | x | x | x | x | x | x | x | x | x | x | x |  | x |  |
|  | Boon & Mitchell 1995 | Ryans 1 Billabong |  | x |  |  | x | x | x | x | x | x | x | x | x | x | x | x |  |  |
|  | Fusé 2016 | La Barrancosa |  | x |  |  | x | x | x | x | x | x | x | x | x | x | x | x | x |  |
